# Assessing the accuracy of automated CT perfusion software in excluding acute stroke: a comparative study of two software packages

**DOI:** 10.3389/fnimg.2025.1613078

**Published:** 2025-10-31

**Authors:** Maximilian Thormann, Maria Faltass, Roland Schwab, Stefan Klebingat, Daniel Behme

**Affiliations:** ^1^Department of Neuroradiology, University Hospital Magdeburg, Magdeburg, Germany; ^2^Department of Nuclear Medicine, Charité Berlin, Berlin, Germany; ^3^Stimulate Research Campus Magdeburg, Magdeburg, Germany

**Keywords:** computed tomography perfusion, acute ischemic stroke, diagnostic accuracy, lacunar infarct, neuroimaging

## Abstract

**Background:**

Computed tomography perfusion (CTP) is frequently used for the rapid assessment of suspected acute ischemic stroke (AIS). However, small lacunar infarcts often remain undetected by automated software, leading to false negatives and additional imaging. We compared the specificity of two commonly used CTP software packages in patients without evidence of stroke on follow-up diffusion-weighted imaging (DWI).

**Methods:**

In this single-center retrospective study, 58 consecutive patients with suspected AIS but negative follow-up DWI–MRI were included. All patients underwent CTP on the same scanner. Perfusion data were processed using (1) syngo.via (Siemens Healthcare) with three parameter settings—A: CBV < 1.2 mL/100 mL, B: additional smoothing filter, and C: rCBF <30%—and (2) Cercare Medical Neurosuite (CMN). Software-reported ischemic core volumes were compared with the MRI findings.

**Results:**

CMN showed the highest specificity, indicating zero infarct volume in 57/58 patients (98.3%). Conversely, all three syngo.via settings produced false-positive ischemic cores, with median volumes ranging from 21.3 mL (setting C) to 92.1 mL (setting A). Only syngo.via setting C reported zero infarct volume in some patients, yet still showed substantial overestimation (maximum 207.9 mL).

**Conclusion:**

Our findings underscore the significant variability in the ability of different CTP software packages to reliably rule out small (lacunar) infarcts. CMN demonstrated good specificity, suggesting that dependable CTP-based stroke exclusion is achievable with advanced post-processing. High specificity could reduce reliance on follow-up MRI in acute stroke pathways if validated, thereby improving resource allocation and patient throughput.

## Introduction

Computed tomography perfusion (CTP) is frequently utilized to assess patients with clinical suspicion of acute ischemic stroke (AIS), as it offers rapid assessment of cerebral perfusion deficits and core size (Koopman et al., 2019; Kim et al., 2024; Hoving et al., 2022; Abdalkader et al., 2023). The two main aims of CTP are to identify patients with large vessel occlusions (LVOs) or distal vessel occlusions (DVOs) suitable for endovascular treatment and to assess tissue viability (Zedde et al., 2023). Perfusion parameters can influence clinical decision-making and shape prognosis. For example, Mei et al. (2025) showed that CTP results and applied thresholds influence triage decisions. Lakhani et al. (2024) demonstrated that CTP parameters predicted poor functional outcomes in AIS.

Lacunar strokes, primarily resulting from small vessel disease or atherothrombotic involvement of the parent artery that occludes a perforating branch, are not commonly addressed in this context. Perfusion changes corresponding to lacunar infarcts are often not detectable on post-processed core-penumbra maps due to smoothing by automated software, which only includes relatively large clusters of hypoperfused pixels in the map (Zedde et al., 2023). The reliability of perfusion maps is therefore low. In current practices, patients with suspected stroke but inconclusive CTP findings often undergo an additional MRI to confirm or exclude ischemia, since diffusion-weighted imaging (DWI) has excellent sensitivity for acute infarction.

However, MRI is costly and often not promptly accessible in hyperacute and acute stroke management. As a more widely available alternative, CTP performance varies significantly due to differences in patient characteristics, spatial/temporal resolution, and post-processing methods (Biesbroek et al., 2013). Notably, CTP maps have lower sensitivity for small lacunar infarcts, which can lead to false-negative results (Biesbroek et al., 2013). For better patient triage and the efficient use of healthcare resources, high specificity of CTP in detecting lacunar infarcts is warranted. Ideally, this would eliminate the need for MRI resources for patients without a detected stroke.

The purpose of this study was to compare the specificity of CTP for ischemic stroke using two commonly used perfusion software packages: syngo.via (Siemens Healthcare, Erlangen, Germany) and Cercare Medical Neurosuite (CMN, Cercare Medical, Aarhus, Denmark), a newly developed automated CTP analysis package.

## Materials and methods

### Study population

In this single-center retrospective analysis, we included all consecutive patients with no detectable stroke on DWI–MRI between January 2021 and November 2022. The sample size reflects all eligible patients during the study period.

The inclusion criteria were as follows:

- Clinical suspicion of acute ischemic stroke.
- Availability of a CTP dataset prior to treatment.
- Follow-up MRI with DWI sequence performed, confirming no infarct.

The exclusion criteria were as follows:

- Patients who received intravenous (IV) thrombolysis between CTP and MRI.
- Severe motion artifacts or poor scan quality on CTP or MRI.
- Failed automated perfusion post-processing.
- Evidence of chronic infarct on FLAIR/DWI in the region of perfusion abnormality.
- Vessel occlusion or stenosis on CT angiography.

**Figure 1:**
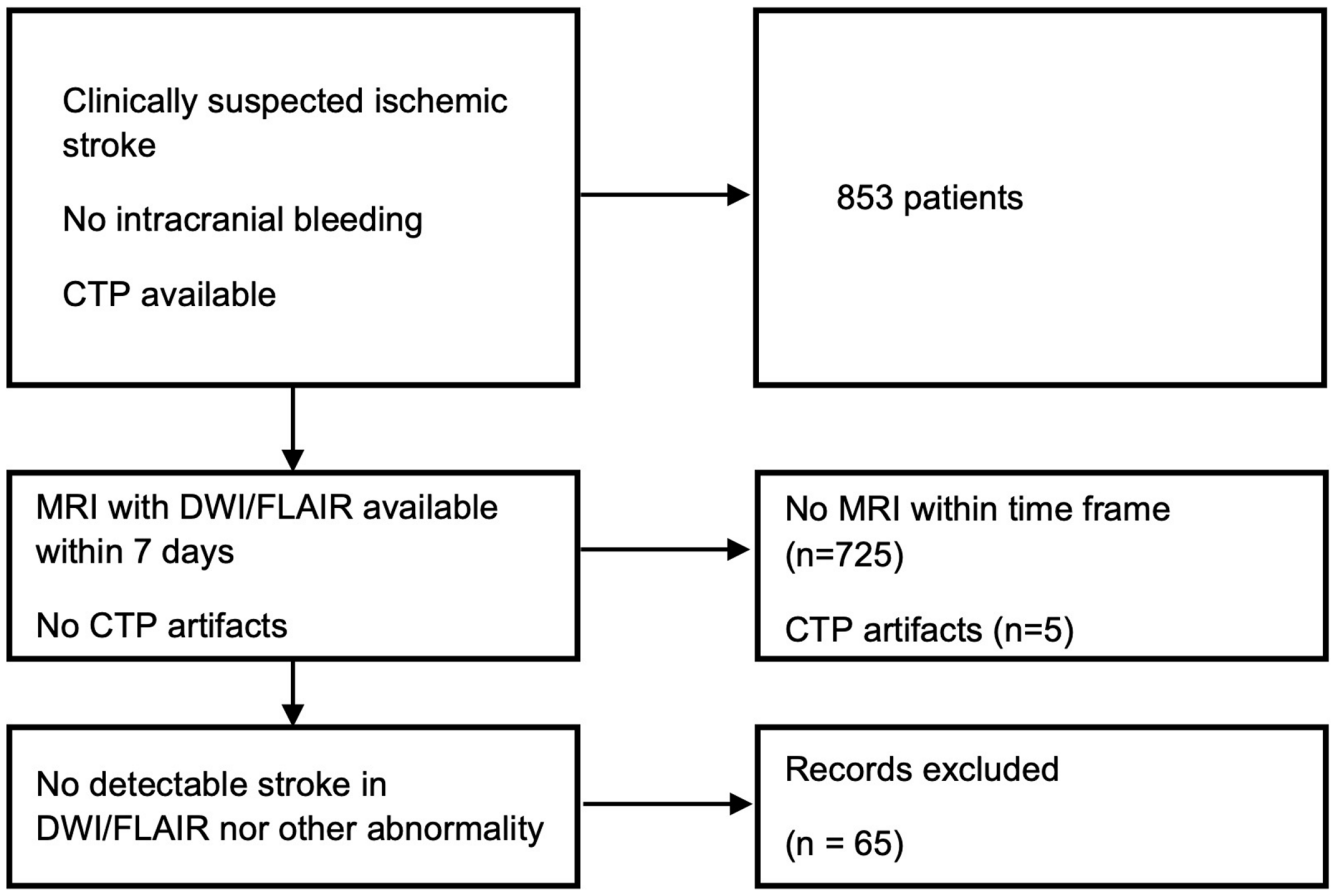
Screening and inclusion flowchart. The final dataset comprised 58 patients.

The patient selection process is detailed in Figure 1.

The study was approved by the local ethics committee.

### Imaging acquisition and post-processing

All CTP scans were performed on the same scanner model (Somatom Definition AS)+ (Siemens Healthcare, Germany). Imaging parameters were as follows: Kernel: T20F, contrast agent: Imeron 300 (Bracco Imaging, Germany), injection rate: 5 mL/s, and start of acquisition: 3 s after injection.

MRI examinations were performed using three scanner models with different field strengths (1.5 T systems Intera, Philips Healthcare, Best, The Netherlands or Magnetom Sola, Siemens Healthcare, Erlangen, Germany; 3.0 T system Achieva, Philips Healthcare, Best, The Netherlands). B-values were 0 and 1,000 s/mm^2^ on all scanners to minimize potential scanner-related variability. The reference standard was no acute infarct on follow-up MRI (DWI ± FLAIR), with a mean delay of 68.1 ± 38.5 h, to minimize DWI-negative early scans. The risk of interval infarction or lesion evolution was minimized by the study design. A false-positive CTP core was defined on a per-patient basis as an automated CTP-identified ischemic core volume >0 with no corresponding acute infarct on follow-up DWI and FLAIR imaging. Conversely, true negatives were defined as patients with no core on CTP and no lesion on DWI.

All perfusion data were post-processed using two fully automated software packages: syngo.via CT Neuro Perfusion (version VB60A) and CMN (version CMN 15.0). All software packages apply automated registration, segmentation, and motion correction. Outputs were recorded as calculated by the software. We focused on infarction core calculations only.

syngo.via CT Neuro Perfusion employs a delay-insensitive deconvolution model and interhemispheric comparison. It determines the lesion side by identifying the highest time-to-drain, using the contralateral side as a reference for relative values. Summary maps emphasize the ischemic core and its volumes.

Adopting the approach by Koopman et al. (2019), analysis with syngo.via was performed using three different settings. In method A, maps were generated based on the software’s default settings. Ischemic core volume was defined as a CBV of <1.2 mL/100 mL. In method B, an additional smoothing filter was added to the same threshold. In method C, the ischemic core was defined as a reduction in cerebral blood flow (CBF) to < 30% compared to healthy brain tissue. The standard software setting for syngo.via corresponded to setting B.

Cercare Medical Neurosuite (CMN) is a recent addition to the market. The software uses a model-based approach to quantify cerebral blood flow (CBF). Instead of relying solely on standard mathematical deconvolution via singular value decomposition (SVD), CMN uses a gamma distribution-based model of the tissue residue function. By capturing the natural variability in transit times through the microvasculature, CMN claims its method provides accurate measurements in low-flow regions, ensuring that ischemic tissue is properly identified and leading to a more precise depiction of the affected area.

A total of two neuroradiologists independently reviewed each software’s perfusion maps to verify the presence or absence of a core lesion as defined by the software and rule out potential matches with older tissue defects. There were no disagreements.

### Clinical data collection

All case-specific and demographic data were obtained from the hospital information system.

### Statistical analysis

Data were presented as mean +/− standard deviation (SD), median (interquartile range (IQR)), and number (percentage), as appropriate. The Wilcoxon test for paired differences was used as a non-parametric test. The root mean square error (RMSE) and mean absolute error (MAE) were calculated to quantify the errors. Furthermore, 95% confidence intervals were estimated using bootstrap resampling (1,000 iterations) of the residuals, without assuming normality. A *p*-value of <0.05 was considered statistically significant.

## Results

**Figure 2:**
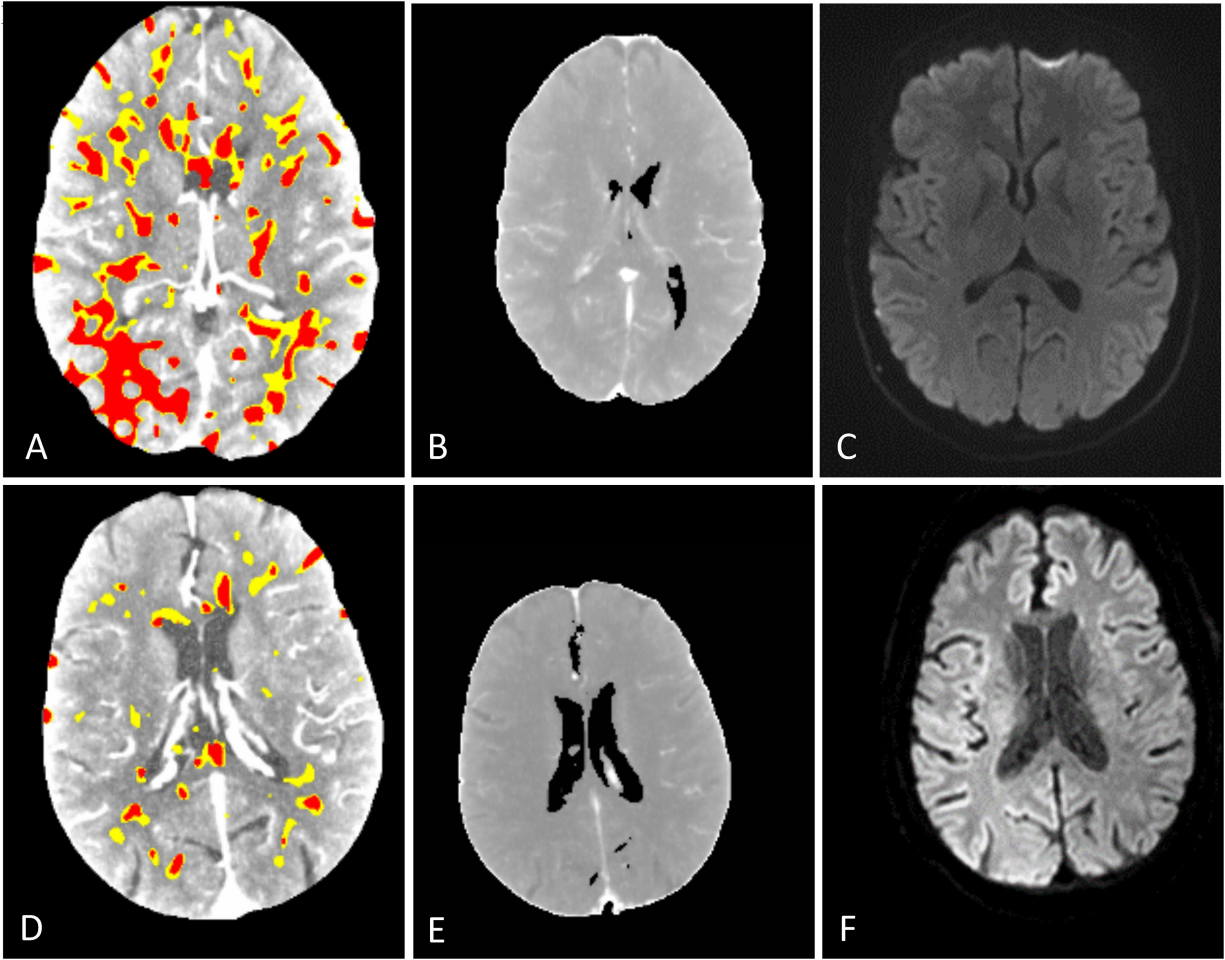
Representative CTP output maps of syngo.via **(A,D)** showing diffuse hypoperfused (yellow) and infarct core (red) areas. CMN hyperperfusion/core maps **(B,E)** show no infarct core. Corresponding MRI DWI **(C,F)** images show no signs of acute infarction

A total of 58 patients met the inclusion criteria for analysis. The average age was 69.6 years (SD 15.7 years). A total of 29 patients were male, and 29 patients were female. In addition, six patients received intravenous thrombolysis. The patients’ baseline characteristics are summarized in Table 1. Extended patient details are given in Supplementary Table 1. The average time from CTP to MRI was 68.12 h (SD 38.53 h). One CTP scan could not be processed by syngo.via B and C due to unknown errors. Exemplary outputs are illustrated in Figure 2.

**Table 1:** Patients’ baseline characteristics.

Characteristic	Value
Total patients	58
Male — *n* (%)	29 (50.0%)
Female — *n* (%)	29 (50.0%)
Intravenous thrombolysis	6 (10.3%)
Age, mean (SD)	69.6 (15.7)
NIHSS, median (min–max)	1 (0–10)
mRS (pre-admission), median (min–max)	0 (0–4)
mRS (at admission), median (min–max)	2 (0–5)
mRS (at discharge), median (min–max)	1 (0–4)

CMN yielded a median ischemic core volume of 0.0 mL (IQR 0.0–0.0 mL, range 0.0–4.7 mL) and a mean of 0.1 mL (SD 0.5 mL) on CTP, matching the 0 mL infarct confirmed on DWI in 57 out of the 58 cases.

By contrast, perfusion analysis with syngo.via indicated substantially higher core volumes in the patients with no infarct:

- Setting A: median 92.1 mL (IQR 66.2–117.8 mL), minimum 29.6 mL, maximum 203.1 mL; mean 94.7 ± 41.4 mL.
- Setting B: median 37.8 mL (IQR 24.6–51.5 mL), minimum 9.5 mL, maximum 93.9 mL; mean 40.8 ± 22.6 mL.
- Setting C: median 21.3 mL (IQR 6.7–40.3 mL), minimum 0.0 mL, maximum 207.9 mL; mean 39.0 ± 31.1 mL.

The results are summarized in Table 2.

**Table 2:** Summary of ischemic core volume measurements by Cercare Medical Neurosuite (CMN) and syngo.via software settings (A, B, C) in the patients without detectable infarcts on follow-up diffusion-weighted imaging (DWI).

Parameter	Vol	CMN	syngo.via A	syngo.via B	syngo.via C
Median (IQR) infarct volume, ml	0.0 (0.0 to 0.0)	0.0 (0.0 to 0.2)	92.1 (66.2 to 117.8)	37.4 (24.6 to 51.5)	21.6 (6.7 to 40.3)
Min/Max infarct volume, ml	0.0/0.0	0.0/4.7	29.6/203.2	9.5/93.9	0.0/207.9
Mean (STD) infarct volume, ml	0.0 (0.0)	0.2 (0.7)	94.7 (41.4)	40.8 (22.6)	29.0 (32.1)
Infarct volume normal distribution (Shapiro–Wilk) (*p*-value)	1.000	0.000	0.088	0.001	0.000
Mean (SD) error, ml		0.2 (0.7)	94.7 (41.4)	40.8 (22.6)	29.0 (32.1)
Mean Absolute Error (CI 95%), ml		0.2 (0.1 to 0.4)	94.7 (84.5 to 105.7)	40.8 (35.0 to 47.1)	29.0 (22.0 to 38.3)
Root Mean Square Error (CI 95%), ml		0.7 (0.2 to 1.1)	103.3 (92.0 to 114.2)	46.5 (40.1 to 52.4)	43.1 (29.1 to 59.8)
Median (IQR) error, ml		0.0 (0.0 to 0.2)	92.1 (66.2 to 117.8)	37.4 (24.6 to 51.5)	21.6 (6.7 to 40.3)
Limits of agreement, ml		−1.1, 1.6	13.5, 176.0	−3.5, 85.1	−34.0, 92.0
Wilcoxon-Signed-Rank		715.500 (*p*-val: 0.230)	0.000 (*p*-val: 0.000)	0.000 (*p*-val: 0.000)	0.500 (*p*-val: 0.000)

**Figure 3:**
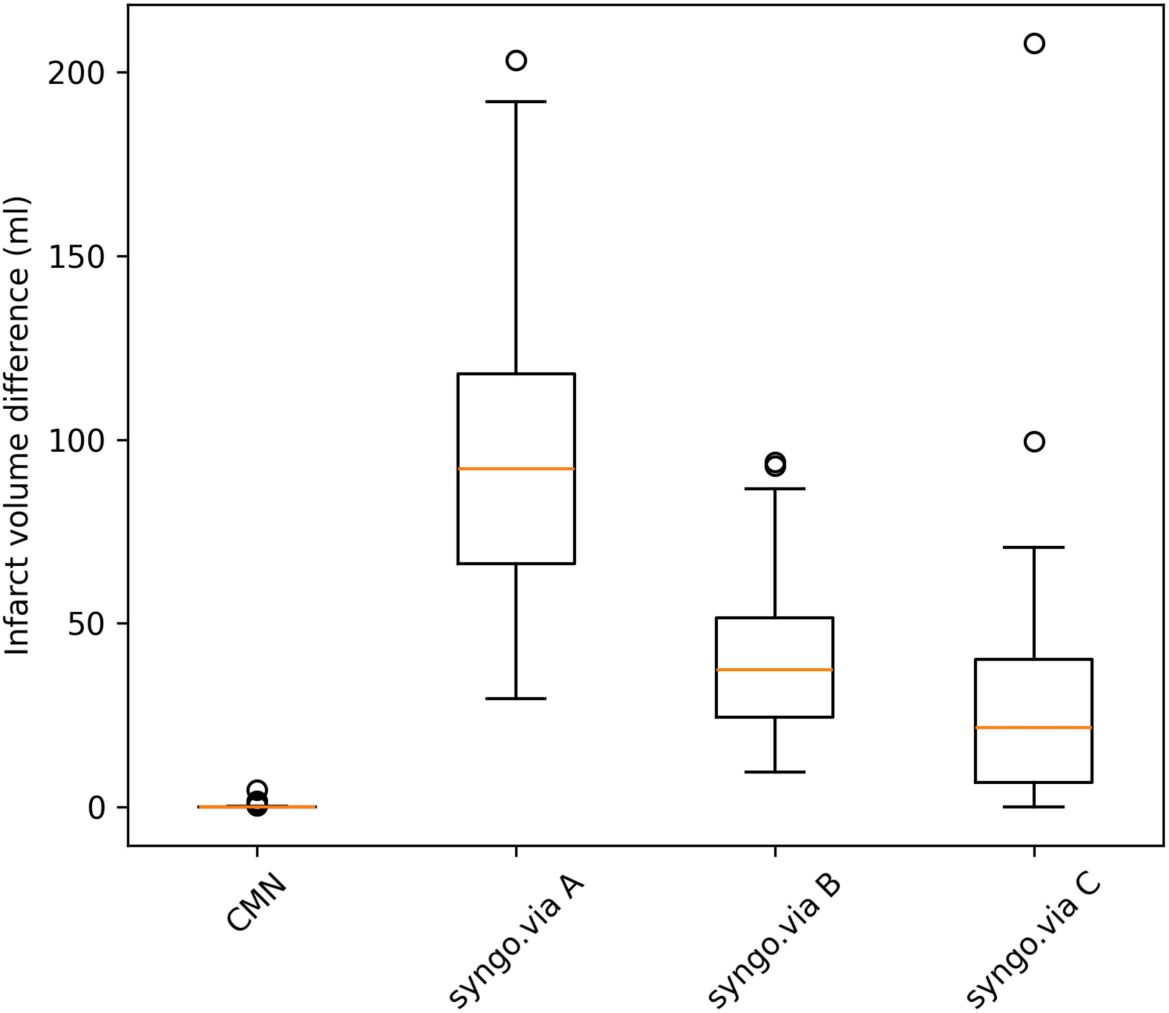
Scatter plot displaying ischemic core volumes calculated by CMN and syngo.via (settings A, B, and C) in a DWI-negative cohort (reference infarct volume = 0 mL). Note the consistent overestimation by syngo.via, potentially risking patient mis-triage. CMN estimates were zero for 57/58 patients.

Figure 3 illustrates the infarct volumes calculated by each software for all 58 patients (true infarct volume by DWI = 0 for all). Notably, syngo.via *setting C* was the only setting to report a 0 mL core volume in one patient, but it still calculated false-positive infarction volumes in most cases. Settings A and B falsely identified a non-zero infarct core in every patient within this no-stroke cohort.

**Figure 4:**
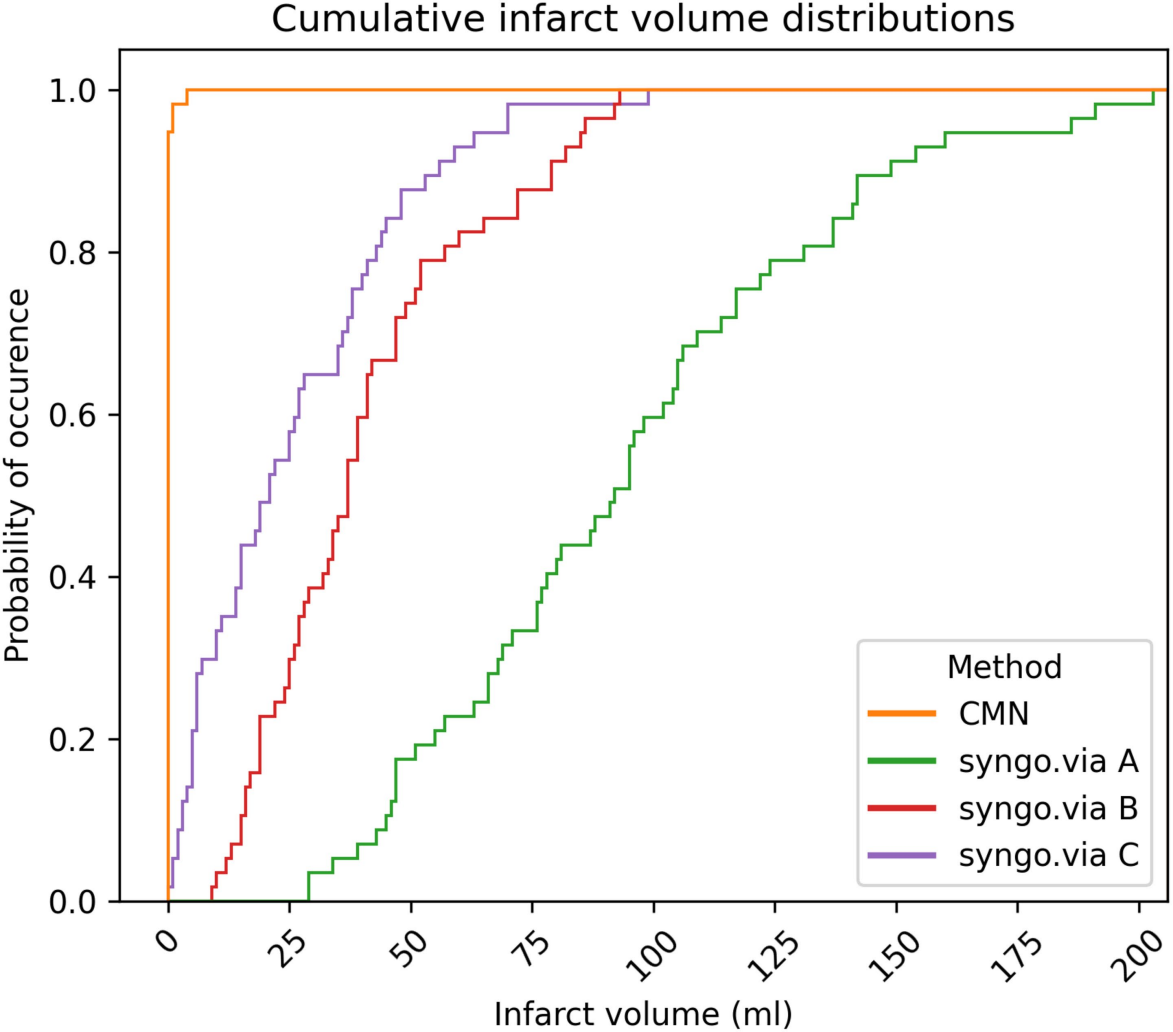
Cumulative distribution of calculated ischemic core volumes by the software packages (CMN and syngo.via settings A, B, C, *n* = 58), illustrating the disparity between the true negative results from CMN and the false-positive core volumes reported by syngo.via.

**Figure 5:**
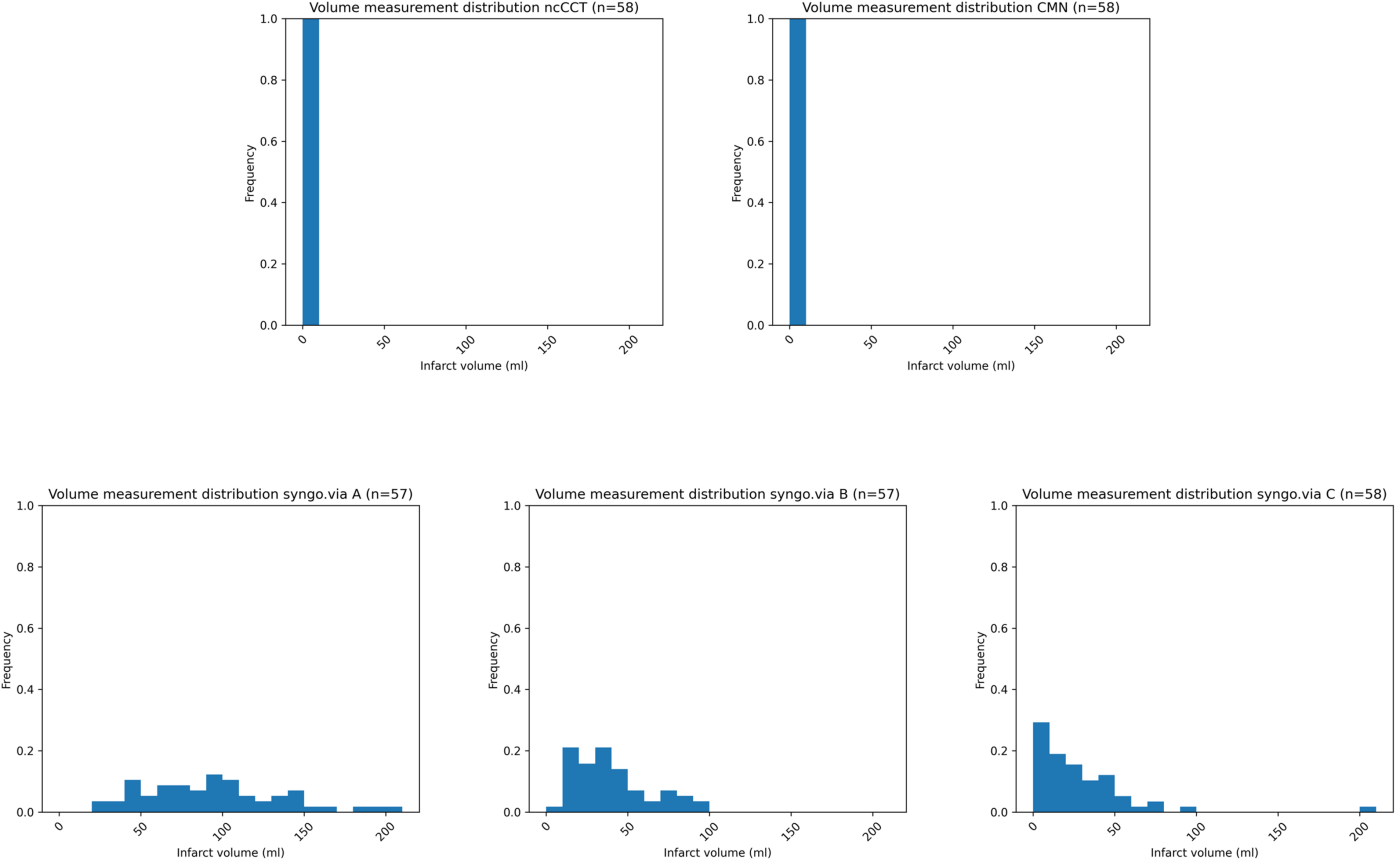
Histograms illustrating the distribution of the calculated ischemic core volumes by Cercare Medical Neurosuite (CMN) and the three parameter settings (A, B, and C) of the syngo.via software. CMN consistently shows no infarct volume, aligning with the reference findings. In contrast, the syngo.via settings (A–C) overestimate infarct volumes, showing significant false-positive rates and potentially leading to patient mis-triage.

Figure 4 illustrates the cumulative distribution of the ischemic core volumes calculated by each software across all included patients. CMN reported negligible infarct volumes, closely matching the DWI findings. Syngo.via overestimated infarct volumes for almost all patients. Figure 5 shows the corresponding histogram analysis comparing the calculated infarct volume distributions.

## Discussion

Our study aimed to determine the specificity of two CT perfusion software packages in acute ischemic stroke. Overall, CMN demonstrated higher accuracy than syngo.via settings in correctly ruling out infarction. Our results indicate that CMN shows high specificity in excluding infarction in a DWI-negative cohort suspected of AIS, while syngo.via calculations are prone to false-positive results across all three settings, as illustrated in Figure 5. As previously shown, applying an additional smoothing filter to syngo.via improved agreement, albeit with reduced accuracy compared to CMN (Koopman et al., 2019).

DWI, while highly sensitive for infarction, can miss a small subset of strokes— reported to be up to 6.8% in the literature—particularly minor strokes and those in the posterior circulation (Edlow et al., 2017; Alkhiri et al., 2024). However, Chalela et al. found that DWI negativity was strongly associated with MRI acquisition within less than 3 h. Similarly, Simonsen et al. (2015) found that patients with DWI-negative stroke underwent MRI scanning sooner after symptom onset compared to patients with DWI-positive infarction (Chalela et al., 2007). Generally, the specificity of DWI is reported to be 95–100% (Abdalkader et al., 2023). We attempted to mitigate the DWI-negativity effect by performing delayed MRI (mean: 68 h after CTP). By this time, even small infarcts are typically detectable on DWI/FLAIR. However, we acknowledge that MRI is not infallible and very small or rapidly resolved infarcts (e.g., in a TIA) could have escaped our detection.

Differences in perfusion processing techniques and threshold definitions across software platforms lead to substantial variability in calculated core volumes (Katyal and Bhaskar, 2021). The specificity of various CTP software packages has been assessed in prior studies. For example, Straka et al. (2010) reported approximately 91% specificity for perfusion-diffusion mismatch detection using RAPID software. Similarly, Benson et al. (2016) found specificity up to 98.7% for lacunar infarct detection with Vitrea.

Despite these generally high specificity values, most automated solutions still struggle to *rule out* small infarcts. In one study by Lin et al. (2009) 13 of 23 infarcts missed on initial CTP were small cortical or lacunar infarcts. Consistently, Biesbroek et al. (2013) found an overall CTP specificity of ~95% for acute ischemic stroke, with nearly two-thirds of false negatives attributable to lacunar infarcts. Similarly, Eckert et al. (2011) noted that lacunar infarcts were the most frequent cause of false-negative findings on multimodal CT imaging. A 2017 meta-analysis by Shen et al. (2017) also confirmed that most stroke cases missed by CTP were of the lacunar subtype.

Lacunar strokes account for approximately 25% of ischemic strokes (Kolominsky-Rabas et al., 2001). However, perfusion deficits from lacunar infarcts are usually not apparent on standard CTP maps, especially for small deep or posterior circulation lesions (Garcia-Esperon et al., 2021). This limitation can lead to underdiagnosis of lacunar stroke on CTP alone (Arba et al., 2020). One reason for these false negatives is the coarse spatial resolution of perfusion CT, along with the tendency of automated algorithms to smooth or average values over small regions. In practice, patients presenting with a lacunar stroke syndrome but a negative CTP study typically undergo additional MRI. MRI with DWI (and FLAIR) has greater sensitivity for detecting recent small infarcts and is generally well-tolerated in patients with lacunar stroke (Wardlaw et al., 2024). However, MRI availability is limited in many stroke centers, and it is time-consuming and resource-intensive. This underscores the value of highly specific CTP software for ruling out lacunar stroke without the need for MRI.

When evaluating patients with suspected acute ischemic stroke (AIS), the ability to confidently exclude ischemia—particularly lacunar infarcts—has significant clinical value. Beyond core and penumbra volumes, recent multi-center and registry analyses indicate that rCBV thresholds and hypoperfusion intensity ratios (HIRs) stratify outcomes in LVO cohorts (Mei et al., 2025; Lakhani et al., 2024). By focusing on specificity in small infarct scenarios, our results complement these selection frameworks by reducing the risk of false positives that could affect treatment decisions.

Robust specificity allows clinicians to avoid unnecessary MRI scans, thereby reducing costs and improving workflow efficiency. In our study, CMN showed a specificity of almost 100%. The difference compared to the syngo.via results in the same cohort was remarkable (Figure 3). syngo.via, across all tested threshold settings, consistently produced false-positive infarct detections. Both syngo.via setting A and setting B wrongly detected strokes in all included patients. Only setting C, using an infarct core threshold of rCBF <30%, correctly detected a 0 mL infarct core in some patients, albeit still with a median core of 21.6 mL and a maximum infarct core detection of 207.9 mL. Conversely, CMN maintained high specificity by consistently reporting negligible core volumes in the patients without true infarcts. CMN’s gamma distribution-based model is designed to capture a wider range of flow patterns in low-flow regions, potentially explaining its higher specificity in excluding small infarcts.

The limited immediate availability of MRI in many stroke centers drives reliance on CTP. Our study indicates that CTP is able to add value to NCCT in the setting of ischemic stroke. Our findings highlight the importance of accurate CTP software in the assessment of acute stroke. In clinical practice, a fast and widely available screening tool that can reliably rule out both large-vessel and small-vessel (lacunar) strokes could streamline patient management. If confirmed, accurate CTP results could decrease the need for follow-up MRI in a large proportion of patients with clinical suspicion of acute stroke. Limited MRI slots could be prioritized for patients for whom advanced imaging is crucial.

While our study uniquely examines specificity in stroke-mimic patients using CMN and syngo.via, direct comparisons of CMN with established reference software packages such as RAPID or MIStar are not yet available. However, CMN’s FDA clearance was based on demonstrated equivalence to RAPID’s analytic approach. Our results suggest that in the specific context of excluding small infarcts, CMN performs strongly. Future studies should confirm if this finding holds true against established platforms in broader populations.

Our study has limitations that need to be acknowledged. It was retrospective in nature and restricted to DWI-negative patients, which allowed us to focus on specificity. Our study did not assess the sensitivity of the included software packages, as AIS-positive cases were excluded by design. While high specificity is desirable to reduce false-positive cores, clinical decision-making requires a balance with sensitivity. Performance in mixed cohorts should be assessed in future studies. The real-world performance of CMN in a standard cohort (with both AIS and non-AIS cases) is not addressed here and should be the subject of future studies. For CMN, we used standard settings only for post-processing. While settings in CMN can be modified, there are no published threshold sensitivity analyses for the software. An extended software-specific threshold analysis was beyond the scope of our study. Our performance estimates reflect the specific software versions tested. Should future vendor updates alter thresholds or deconvolution approaches, the generalizability of our findings may change and would warrant re-evaluation. Follow-up MRI DWI was performed on three different scanners with different field strengths. Scanner heterogeneity may affect small lesion conspicuity and permit interval changes. The average interval to follow-up MRI was long. A total of six patients received IV thrombolysis, potentially influencing the MRI results. Finally, our sample size, while focused on a specific no-stroke cohort, was modest, reflecting the number of eligible patients during the study period.

Our study underscores the value of a highly specific CTP software package for excluding acute stroke, including lacunar subtypes, thereby reducing unnecessary MRI utilization. In our cohort, CMN demonstrated a specificity of almost 100% for small strokes. Despite improvements in various commercial CTP solutions, substantial variability persists, and software selection may have a direct impact on patient triage, treatment decisions, and overall stroke care costs. Accurate CTP core quantification and reliable exclusion of small infarcts will remain critical. Future studies should test integrated pipelines that pair high-specificity core estimation with venous outflow/collateral metrics and mismatch indices, as recently suggested (Barghash et al., 2025), assessing whether combined models enhance accuracy and clinical outcome prediction.

## Data Availability

The raw data supporting the conclusions of this article will be made available by the authors, without undue reservation upon reasonable request.
