# Supplementary material for: Assessing the accuracy of automated CT perfusion software in excluding acute stroke: a comparative study of two software packages
Source: Front Neuroimaging. 2025 Oct 31;4:1613078. doi: 10.3389/fnimg.2025.1613078 (PMC12615249; doi:10.3389/fnimg.2025.1613078)
Supplement: Supplementary file 1 [file Table_1.docx]

**Supplementary Table 1. Characteristics of included patients**

| **Patient Nr.** | **Male/Female** | **Age** | **NIHSS** | **mRS pre admission** | **mRS at admission** | **mRS at discharge** | **i.v. Thrombolysis** | **Preexisting small tissue defects** | **Diagnosis** |
| --- | --- | --- | --- | --- | --- | --- | --- | --- | --- |
| 1 | F | 59 | n/a | n/a | n/a | n/a | 1 | 0 | Sepsis |
| 2 | M | 59 | 0 | 0 | 4 | 2 | 1 | 0 | Unclear/TIA |
| 3 | F | 79 | n/a | n/a | n/a | n/a | 0 | 1 | Epilepsy |
| 4 | M | 63 | n/a | n/a | n/a | n/a | 0 | 1 | Epilepsy/Encephalopathy |
| 5 | M | 69 | 3 | 0 | 4 | 2 | 0 | 1 | Unclear/TIA |
| 6 | F | 68 | 1 | 0 | 2 | 2 | 0 | 0 | Unclear/TIA |
| 7 | F | 85 | n/a | n/a | n/a | n/a | 0 | 0 | Normal pressure hydrocephalus (NPH)/Encepalopathy |
| 8 | M | 80 | 1 | 2 | 2 | 2 | 0 | 1 | TIA |
| 9 | F | 91 | n/A | n/a | n/a | n/a | 0 | 0 | Epilepsy |
| 10 | M | 32 | 8 | n/a | n/a | n/a | 0 | 0 | Unclear/Encephalitis |
| 11 | M | 78 | 1 | 0 | 0 | 0 | 0 | 0 | TIA |
| 12 | F | 42 | 0 | 0 | 0 | 0 | 0 | 0 | Unclear |
| 13 | F | 58 | 1 | 0 | 2 | 0 | 0 | 1 | Unclear/TIA |
| 14 | F | 77 | n/a | n/a | n/a | n/a | 0 | 1 | Delirium |
| 15 | F | 68 | 0 | 0 | 0 | 0 | 0 | 0 | Unclear/TIA |
| 16 | m | 62 | 0 | o | 4 | 0 | 0 | 0 | Unclear/TIA |
| 17 | f | 50 | 0 | 0 | 1 | 0 | 0 | 0 | Unclear/TIA |
| 18 | f | 80 | n/a | n/a | n/a | n/a | 0 | 0 | Dementia |
| 19 | f | 50 | 0 | n/a | n/a | n/a | 0 | 0 | Neuroborreliosis |
| 20 | f | 86 | 6 | 4 | 5 | 4 | 0 | 0 | Hypotension |
| 21 | m | 68 | 1 | 1 | 1 | 1 | 0 | 0 | Unclear/TIA |
| 22 | m | 82 | 1 | n/a | n/a | n/a | 0 | 0 | Epilepsy |
| 23 | m | 80 | 0 | n/a | n/a | n/a | 0 | 0 | Idiopathic abducens nerve palsy |
| 24 | f | 81 | 7 | n/a | n/a | n/a | 0 | 0 | Myeloradiculopathy |
| 25 | m | 53 | 1 | 0 | 0 | 0 | 0 | 0 | Unclear/TIA |
| 26 | m | 70 | n/a | n/a | n/a | n/a | 0 | 0 | Vertigo |
| 27 | f | 74 | 0 | n/a | n/a | n/a | 0 | 0 | Hepatic encephalopathy |
| 28 | m | 69 | n/a | n/a | n/a | n/a | 0 | 0 | Intracranial space-occupying lesion |
| 29 | m | 53 | 0 | n/a | n/a | n/a | 0 | 0 | Intracranial space-occupying lesion |
| 30 | m | 87 | 9 | 2 | 4 | 2 | 0 | 0 | Unclear/TIA |
| 31 | m | 77 | n/a | n/a | n/a | n/a | 0 | 0 | Unclear/TIA |
| 32 | m | 54 | n/a | n/a | n/a | n/a | 0 | 0 | Epilepsy |
| 33 | f | 56 | n/a | n/a | n/a | n/a | 0 | 0 | Migraine |
| 34 | f | 84 | n/a | n/a | n/a | n/a | 0 | 1 | Epilepsy |
| 35 | m | 46 | 1 | 0 | 0 | 0 | 0 | 0 | TIA |
| 36 | m | 75 | 2 | 0 | 2 | 0 | 1 | 1 | Unclear/TIA |
| 37 | m | 66 | 1 | 0 | 3 | 2 | 1 | 0 | Unclear/TIA |
| 38 | f | 78 | n/a | n/a | n/a | n/a | 0 | 0 | Epilepsy |
| 39 | m | 60 | n/a | n/a | n/a | n/a | 0 | 1 | Unclear/TIA |
| 40 | f | 88 | 10 | 0 | 4 | 0 | 0 | 0 | Vertigo |
| 41 | f | 90 | 9 | 0 | 3 | 0 | 1 | 0 | Unclear/TIA |
| 42 | m | 82 | n/a | n/a | n/a | n/a | 0 | 0 | Unclear/TIA |
| 43 | f | 82 | n/a | n/a | n/a | n/a | 0 | 0 | Epilepsy |
| 44 | f | 80 | 0 | n/a | n/a | n/a | 0 | 0 | Unclear/TIA |
| 45 | f | 40 | 1 | 0 | 2 | 2 | 0 | 0 | Unclear/TIA |
| 46 | f | 84 | n/a | n/a | n/a | n/a | 0 | 0 | Epilepsy |
| 47 | m | 81 | 0 | n/a | n/a | n/a | 0 | 0 | Dementia |
| 48 | f | 78 | 1 | 0 | 2 | 2 | 0 | 1 | Unclear/TIA |
| 49 | m | 88 | n/a | n/a | n/a | n/a | 0 | 1 | Epilepsy |
| 50 | m | 39 | n/a | n/a | n/a | n/a | 0 | 0 | Epilepsy |
| 51 | m | 65 | 1 | n/a | n/a | n/a | 0 | 0 | Epilepsy |
| 52 | f | 75 | 0 | 0 | 0 | 0 | 0 | 0 | Unclear/TIA |
| 53 | m | 82 | n/a | n/a | n/a | n/a | 0 | 0 | Epilepsy |
| 54 | m | 66 | 6 | 0 | 3 | 1 | 1 | 0 | Unclear/TIA |
| 55 | m | 67 | 4 | 0 | 3 | 2 | 0 | 1 | Unclear/TIA |
| 56 | f | 88 | n/a | n/a | n/a | n/a | 0 | 0 | Rotational vertigo |
| 57 | f | 89 | 1 | 2 | 3 | 2 | 0 | 1 | TIA |
| 58 | f | 27 | 3 | n/a | n/a | n/a | 0 | 0 | Migraine |
